# Clinical management of herpes simplex virus infections: past, present, and future

**DOI:** 10.12688/f1000research.16157.1

**Published:** 2018-10-31

**Authors:** Richard Whitley, Joel Baines

**Affiliations:** 1 Department of Pediatrics, Microbiology, and Medicine, University of Alabama at Birmingham Children’s Hospital, Birmingham, AL, 35233, USA; 2 Department of Pathobiology, School of Veterinary Medicine, Louisiana State University, Baton Rouge, LA, 70803, USA

**Keywords:** Herpes simplex virus, antiviral compounds, Herpes encephalitis, CRISPR/Cas9, vaccine, HSV-1, HSV-2

## Abstract

Infection with herpes simplex virus (HSV) types 1 and 2 is ubiquitous in the human population. Most commonly, virus replication is limited to the epithelia and establishes latency in enervating sensory neurons, reactivating periodically to produce localized recurrent lesions. However, these viruses can also cause severe disease such as recurrent keratitis leading potentially to blindness, as well as encephalitis, and systemic disease in neonates and immunocompromised patients. Although antiviral therapy has allowed continual and substantial improvement in the management of both primary and recurrent infections, resistance to currently available drugs and long-term toxicity pose a current and future threat that should be addressed through the development of new antiviral compounds directed against new targets. The development of several promising HSV vaccines has been terminated recently because of modest or controversial therapeutic effects in humans. Nevertheless, several exciting vaccine candidates remain in the pipeline and are effective in animal models; these must also be tested in humans for sufficient therapeutic effects to warrant continued development. Approaches using compounds that modulate the chromatin state of the viral genome to suppress infection and reactivation or induce enhanced antiviral immunity have potential. In addition, technologies such as CRISPR/Cas9 have the potential to edit latent viral DNA in sensory neurons, potentially curing the neuron and patient of latent infection. It is hoped that development on all three fronts—antivirals, vaccines, and gene editing—will lead to substantially less HSV morbidity in the future.

## Introduction

Herpes simplex virus (HSV) infections have been reported since ancient Greek times ^1, 2^. During the 20th century, the clinical manifestations of these infections were broadly reported in the medical literature. In particular, life-threatening disease, such as neonatal HSV infections and herpes simplex encephalitis, appeared in the literature for the first time in the 1930s. Several fundamental early observations provide the basis for today’s understanding of HSV. First, once infected, an individual can have recurrences in spite of both humoral and cell-mediated immune responses (leading to the recognition that the virus establishes latency and may recur upon various provocative stimuli to produce disease). Second, the differentiation between HSV-1 and HSV-2 was clearly recognized. Historically, HSV-1 was associated with infection above the belt, namely the mouth and eye, whereas HSV-2 was attributed to infections below the belt, specifically genital herpes. However, in contemporary times, there is significant overlap between the sites of HSV infection with an ever-increasing proportion of genital herpes caused by HSV-1. In the US, over 50% of adults are seropositive for HSV-1 and about 15% of those who are sexually active are infected by HSV-2 ^3– 6^. Infections caused by these viruses are, for the most part, benign. Third, person-to-person transmission, particularly in boarding houses and among sexual partners, was reported.

Only toward the end of the 20th century was there a ballooning of knowledge regarding the epidemiology, pathogenesis, virulence, and fundamentals of latent infection. The application of two important tools allowed major insights into pathogenesis, namely type-specific serology and polymerase chain reaction (PCR). Furthermore, antiviral therapy, particularly acyclovir, became routinely available, marking the beginning of the management of HSV disease. During the 21st century, important new observations relevant to the pathogenesis have been identified. Furthermore, the identification of novel antiviral approaches as well as the application of contemporary technology, such as CRISPR/Cas9, should prove of value moving forward. This review will summarize advances which have been realized within the past several years and outline promising future approaches to prevent and decrease HSV-mediated disease.

## Neonatal herpes simplex virus infection

Neonatal HSV infections are among the most devastating of all those caused by this virus ^7^. Recent analyses of large-population databases provide insight into the incidence of disease in the US. Whereas the incidence is low, namely 5.24 cases per 10,000 live-births in the US, the potential morbidity and mortality from this infection remain high; adjusted mortality is about 4% in spite of antiviral therapy ^6, 8^. For reasons that are unclear, the incidence is much lower in other countries of the world.

There are three forms of neonatal HSV infection: disease localized to the skin, eye, and mouth (SEM); encephalitis; and disseminated disease ^1^. The development and licensure of acyclovir for the treatment of life-threatening HSV disease have had a major impact on clinical outcomes. About 45% of babies will have SEM disease for which there is no attributable mortality; however, some of these babies can develop intellectual impairment for reasons noted below.

About 35% of affected babies will have central nervous system (CNS) disease and the remaining will have disseminated multi-organ disease. Mortality is the highest with disseminated disease, about 40%, whereas mortality with CNS disease is only 5% ^7, 9^.

Several recent advances in our understanding of neonatal HSV disease are particularly noteworthy. First, utilization of PCR has allowed the diagnosis of CNS disease without either brain biopsy or culture of the cerebrospinal fluid (CSF) ^10^. More importantly, of those babies who appear to have disease localized to the SEM, about 10% have detectable HSV DNA within the CSF at the time of clinical presentation. The cytology of their CSF was entirely normal, implying that they had asymptomatic infection of the CNS. Indeed, this group of children ultimately developed neurologic impairment. Thus, it has become standard of care to assess the CSF for the presence of HSV DNA at presentation. If HSV DNA is detected, these babies should be managed with a longer course of acyclovir (21 days) than if disease were simply limited to the SEM (14 days) ^11^.

Second, PCR analyses that were performed at the beginning and conclusion of treatment identified several important factors. For babies with CNS infection, CSF PCR remained positive for HSV DNA after a full course of antiviral therapy, namely 14 days, in 40% of newborns. This finding is of significant concern and implies that treatment should be extended, as is now the case, to 21 days. Equally importantly, those babies who had HSV-2 infections of the CNS were more likely to remain PCR-positive in the CSF than those who were infected by HSV-1. This finding mirrored the morbidity such that those babies with HSV-2 infections had a much more severe neurologic outcome than those babies with HSV-1. Lastly, PCR positivity at the conclusion of antiviral therapy predicted long-term outcome, including mortality.

With babies remaining PCR-positive at the completion of intravenous antiviral therapy, an important question was, “Do these children have low-grade persistent infection of the central nervous system?” In the absence of a direct way to determine such a finding, long-term suppressive therapy with oral acyclovir was evaluated in a placebo-controlled study ^12^. The data from this study indicate that those who received oral acyclovir for six months following intravenous drug had a significantly better neurologic outcome, as determined by the Bayley Developmental Scale III, than those who received the counterpart placebo. This is not an insignificant finding, as it implies that HSV has the ability to replicate in the brain at low levels and may, as a consequence, result in long-term neurologic damage. With the management approach of six months of suppressive therapy, the number of children who return to normal function can be increased significantly. Suppressive therapy has recently become the management of choice as recommended by the American Academy of Pediatrics Red Book committee ^11^.

As no vaccine is immediately on the horizon (see ‘Vaccines’ section below), alternative methods of preventing neonatal herpes need to be developed. Rapid PCR assays deployed in the delivery suite to determine whether a woman is excreting virus at the time of delivery are being evaluated with the opportunity to administer antiviral therapy to the baby delivered through an infected birth canal. These studies remain experimental and should be completed by 2020. In the meantime, the American Academy of Pediatrics has recommended that babies delivered through an infected birth canal be managed in a definitive prospective fashion. Namely, if the mother has an established recurrent infection, surface swabs are obtained for PCR analysis and for cultures. If the PCR is positive, CSF would need to be examined and intravenous acyclovir therapy instituted. On the other hand, if the mother has a confirmed primary infection, acyclovir for a minimum of 14 days is recommended, following cutaneous swab evaluation for accretion of virus and CSF assessment. Hopefully, this approach will decrease the incidence of neonatal herpes simplex infection.

## Herpes simplex encephalitis

Although antiviral therapy was proven to decrease mortality from herpes simplex encephalitis with the institution of vidarabine therapy in the late 1970s, it was not until much later (1986) that acyclovir was proven to be beneficial in the treatment of biopsy-proven herpes simplex encephalitis ^13^. Significant knowledge has increased our understanding of the diagnosis, treatment, and management of herpes simplex encephalitis. First, the overall incidence of herpes simplex encephalitis has not changed significantly since the early 1980s (1 in 100,000 to 150,000 individuals) ^14^. In the absence of a formal reporting system, it is unlikely that greater precision in incidence will be achieved. Second, and more importantly, the application of PCR for the detection of HSV DNA in the CSF provided the gold standard for the diagnosis of herpes simplex encephalitis, replacing brain biopsy as the legitimate diagnostic ^15, 16^. Importantly, the sensitivity and specificity of PCR were established in comparison with biopsy in biopsy-proven disease versus those with other diseases. However, although PCR is the gold standard for diagnosis, it should be recognized that there is significant variability in test performance between one laboratory and another. Appropriate controls must be employed in order to obtain a reliable and reproducible result. Second, because of the high morbidity and mortality of herpes simplex encephalitis (mortality of about 20% six months after disease onset), the early institution of intravenous acyclovir therapy has been adopted across the US. Usually but not uniformly, empiric treatment is provided to patients who present with findings compatible with herpes simplex encephalitis ^17^. Specifically, those patients with fever, altered mentation, and CSF abnormalities with or without focal neurologic findings are started on acyclovir while PCR studies on the CSF are being performed. It is unclear whether the early institution of such antiviral therapy has had a major impact on the morbidity and mortality of the disease.

Since the original descriptions of morbidity with the first clinical trials of acyclovir, an understanding of outcome has been developed through reviews and summaries of patients that total over 200 individuals with proven herpes simplex encephalitis ^18, 19^. Overall, acute 30-day mortality has been reported to be as low as 5 to 10% but increases to about 20% by day 180. Of the remaining population, 20% have severe neurologic sequelae, 20% have moderate neurologic sequelae, and 40% are left minimally impaired.

Recently, a placebo-controlled study tested the hypothesis that long-term oral valacyclovir administration would improve neurologic outcome for those with proven disease, as oral acyclovir did for neonatal HSV infection ^20^. However, when administered at a dose of 2 grams three times a day for three months, valacyclovir did not improve outcome as compared with placebo. Nevertheless, the study provided, for the first time, data that defined the long-term functional status (~two years) after the onset of acute disease. It also demonstrated that significant and dramatic neurologic improvement occurred regardless of receiving drug or placebo during this two-year period, but especially in the first six months, using very sophisticated neurologic evaluations. Specifically, a Mattis Dementia Rating Scale and a Mini-Mental State Examination were applied to 79 patients with confirmed herpes simplex encephalitis. At baseline (day 30), 36% were assessed as having moderate/severe impairment with both tests. Conversely, 64% were assessed as having mild impairment. At 6-, 12-, and 24-month assessments, the percentage of patients with moderate/severe impairment decreased to 16, 12, and 10, respectively, for both tests. Thus, 90% of patients were functional with no or mild impairment.

As noted above, the long-term administration of valacyclovir to patients with herpes simplex encephalitis was instituted with the presumption that chronic replication could occur in the CNS as presumably is the case in babies with neonatal herpes caused by HSV-2. However, herpes simplex encephalitis in older children and adults is routinely caused by HSV-1. Thus, the implication is that HSV-1 does not have the propensity to replicate chronically in the brain as occurs with HSV-2.

Much remains to be improved in the management of this disease. Drugs that cross the blood–brain barrier as well as combination therapies should be considered for future studies of herpes simplex encephalitis. Perhaps only then will mortality and morbidity be further reduced.

## Resistance

With the advent of antiviral therapy for herpesvirus infections, the concern for resistance has become one of paramount importance. In the early 1980s, acyclovir was the first of the nucleoside analogs having an acceptable safety profile that was licensed for the treatment of HSV infections. Medication could be applied topically, administered intravenously, or taken orally. Since then, prototypic drugs for the treatment of these infections include famciclovir (the prodrug of penciclovir) and valacyclovir (the prodrug of acyclovir). All of these drugs must be phosphorylated in order to be active. Upon entry of the drug into infected cells, the first phosphorylation step is performed by the virus-encoded thymidine kinase. Two additional phosphorylation steps are then mediated by host cellular kinases. Ultimately, acyclovir triphosphate is a substrate for viral DNA polymerase and becomes a DNA polymerase chain terminator because of the lack of a 3′-hydroxyl group. Penciclovir triphosphate, however, allows limited chain elongation because of a 3′-hydroxyl group on its acyclic side chain. These medications have become the mainstay for the management of both HSV and varicella zoster virus infections in the immune-competent as well as the immune-compromised host.

Short-term administration of any of these medications is very rarely associated with the development of resistance. However, long-term administration has been associated with the development of resistance, particularly in the immunocompromised host. In large part, this is due to prolonged high levels of replication in the immunocompromised host that are consequential to a compromised immune response.

For example, screening surveys of immunocompetent patients would indicate that the prevalence of HSV resistance is less than 1% ^21, 22^. However, in the immunocompromised host, the prevalence varies between about 4 and 10%. In HIV-infected patients, particularly those with a low CD4 count, resistance occurs in about 7% of patients. In solid organ and hematopoietic stem cell transplant recipients, the prevalence of resistance is consistently higher, exceeding 10% ^23^. In such cases, persistent and severe disease have been reported. Thus, as will be noted below, alternative strategies for managing these patients are mandatory ^24, 25^.

As noted above, these nucleoside analogs require intracellular phosphorylation by herpes simplex thymidine kinase. Thus, mutations in the *UL23* gene that encodes viral thymidine kinase can arise and confer resistance. Three different phenotypes have been reported: thymidine kinase–negative mutants, which lack enzymatic activity; low thymidine kinase–producing mutants that express very low levels of this enzyme; and thymidine kinase–altered isolates, which are substrate-specific mutants. The majority of mutants encountered in humans are those that are thymidine kinase–deficient. Most cases of acyclovir resistance result from mutations of the *UL23* gene that result in frame shifts in the coding sequence. DNA polymerase mutations are encountered significantly less frequently than thymidine kinase mutants but can confer drug resistance as well ^26^.

When resistance is suspected on the basis of observations of progressive lesions that develop during appropriate antiviral therapy, either genotypic or phenotypic testing should be performed. In some cases, the resistance can be overcome by administering intravenous acyclovir at higher doses. However, this is of only limited utility. As a consequence, alternative therapeutics may be required. At present, only two such therapeutics are approved by the US Food and Drug Administration: foscarnet and cidofovir. Foscarnet is a pyrophosphate analog that inhibits viral DNA polymerase by mimicking the structure of pyrophosphate. As such, it does not require phosphorylation for activation. Rarely, foscarnet-resistant isolates have been obtained from individuals with AIDS. The alternative drug currently available is cidofovir. Cidofovir is an acyclic nucleoside phosphonate. The drug does not require virus-encoded thymidine kinase to be activated. Mutations conferring resistance to cidofovir have been mapped to certain domains of the DNA polymerase. Both foscarnet and cidofovir are associated with toxicity and therefore must be used with caution ^24^. Thus, it is obvious that alternative therapeutic approaches are required.

## Further unmet medical needs

### Antivirals

In spite of the advances in the management of HSV infections with nucleoside analogs, improved therapeutics with alternative mechanisms of actions and enhanced bioavailability are required. Many patients have recurrent corneal disease caused by HSV, and reviews on management have been published recently ^27, 28^. Viral replication and the potentially sight-threatening inflammation and scarring that accompanies it are often kept in check by long-term acyclovir therapy. Long-term administration, however, can lead to a substantial level of drug resistance in viruses isolated from the affected eye ^29^. New antiviral drugs alone or in combination with acyclovir should be developed to help control such infections and to diminish the possibility of resistance.

Two medications are being evaluated for the management of systemic HSV infections: pritelivir and brincidofovir. Pritelivir is a helicase primase inhibitor that plays an essential role in HSV DNA replication ^30– 32^. Pritelivir is extremely active in cell culture and has been shown in two clinical trials to have significant activity against genital HSV infections. To date, therapy has been limited to the short-term treatment of genital herpetic infections as opposed to long-term suppressive therapy. Further long-term toxicity studies are in progress to determine acceptability of long-term suppressive administration. Pritelivir is currently being evaluated for effectiveness against acyclovir-resistant HSV infections in the immunocompromised host.

Brincidofovir, a lipophilic derivative of cidofovir, has a mechanism of action similar to that of cidofovir but does not have the associated nephrotoxicity. The medication was originally developed for the management of human cytomegalovirus infections in hematopoietic stem cell transplant recipients ^33^. However, gastrointestinal tract toxicity was noted and therefore the drug is no longer being evaluated for this indication. It is unclear whether this medication will ultimately be available for administration to humans under such circumstances. Nevertheless, the medication does have activity against acyclic nucleoside–resistant viruses.

The development of new therapeutics for HSV infections is clearly impeded by the lack of interest on the part of the pharmaceutical industry. Because of the high safety of drugs such as acyclovir, valacyclovir, and famciclovir, the market need for additional drugs is significantly reduced from the perspective of most pharmaceutical companies.

Many lessons have been learned from the management of HIV infection and the treatment of hepatitis C virus infections. The most salient of these lessons is the synergistic activity of combination therapies. Such an approach needs to be developed in the management of life-threatening HSV infections, particularly of the newborn and of the CNS. Experimental data in animal models unequivocally show synergy when acyclovir and brincidofovir are combined in the treatment of murine models of neonatal HSV infections; synergy has also been noted upon combination of pritelivir and acyclovir in the management of murine encephalitis models. It is hoped that, within a reasonable period of time, these medications can be brought into human studies despite the potential toxicity of the former and inability to administer the latter in the long term ^34^.

### Vaccines

The goal of HSV therapeutic vaccines would be to reduce the severity of symptoms, accelerate healing of lesions, and reduce virus shedding and infectivity to other individuals. Preventative vaccines might also preclude symptoms of disease induced by wild-type virus and reduce or prevent virus shedding. For HSV, vaccination would be particularly useful in women to reduce transmission to newborns during birth and in the general population to reduce recurrent ocular disease and to reduce transmission to sexual partners. An example of a successful preventative herpes virus vaccine is the varicella zoster vaccine, which has been shown to reduce shedding and severity of varicella (chicken pox) in children ^35^.

Despite many attempts, neither a therapeutic nor preventive vaccine exists for HSV-1 or -2. Although all vaccines that have been investigated thus far stimulate virus-specific immune responses and reduce mortality and virus shedding in animals, they have ultimately yielded disappointing results in human trials, leading to termination of vaccine development. The most recent of these are as follows.

In June 2018, a VICal vaccine trial “did not meet proper end point” in diminishment of HSV-2–induced lesions and the VICal HSV-2 program was terminated. This vaccine comprises plasmids encoding HSV-2 viral proteins glycoprotein D, VP11/12 encoded by UL46, and UL47-encoded VP13/14 formulated with cationic lipid–based adjuvant Vaxfectin ^®^^36^. Although the vaccine was well tolerated during the human trial and was previously shown to be effective against recurrent lesions in animal models, the magnitude of the effect in the test population—HSV-2–infected individuals who reactivated relatively frequently (four to nine times per year)—did not warrant further investment.

Second, the company Genocea (Cambridge, MA, USA) will reportedly no longer singularly develop its GEN-003 vaccine after results from a partially conducted phase III trial revealed only limited clinical efficacy. This vaccine comprises HSV-2 gD lacking its transmembrane domain and a truncated form of infected cell polypeptide 4 (ICP4) formulated in a Matrix M-2 adjuvant. Vaccination with GEN-003 reduced shedding and severity of lesions in animal models and reduced HSV-2 shedding in human patients ^37^.

Third, an attenuated HSV-2 vaccine lacking sequences encoding the viral transcriptional transactivator ICP0 was tested in animals and humans ^38^. Although the vaccine reduced mortality and viral shedding in mice ^39^, controversial trials in humans were conducted in the absence of full institutional review board permission and are being investigated. Anecdotes from vaccinees claiming both efficacy and severe side effects have been reported.

Despite these setbacks, several new vaccine candidates continue to show promising results in animal models:

1. An HSV-2 vaccine lacking two genes ( *UL5* and *UL29*) essential for DNA replication has been shown to induce specific immune responses and significantly reduce mortality, viral shedding, and duration of lesions in virus-challenged animals ^40^. This vaccine is unable to produce infectious virus, thereby limiting spread from cell to cell. It poses a potential advantage over subunit vaccines because it allows presentation of viral antigens in the context of major histocompatibility complex (MHC) molecules in infected cells, thus boosting T-cell responses.
2. The vaccine VC2 contains deletions of sequences within gK and *UL20* that render the virus unable to enter axons. However, VC2 replicates well in epithelial cells allowing its propagation in epithelial cell lines ^41– 43^. Precluding viral entrance into axons is a potentially important feature as it should prevent establishment of latent infection in the bodies of sensory neurons. VC2 has been shown to induce protection of mice from lethal challenge and to induce neutralizing antibodies in rhesus macaques.
3. A vaccine comprising the HSV-2 glycoproteins C, D, and E has been shown to induce neutralizing antibodies against gD and antibodies that reduce the immune evasion activities of gC and gE ^44^. Vaccination of macaques and guinea pigs reduced severity of lesions upon challenge.

Whether any of these promising leads will ultimately generate an effective HSV-1 or HSV-2 vaccine will require demonstration of substantial reduction of lesions in human clinical trials. In the past, this has been a difficult hurdle to overcome.

### Gene editing

Latent infection with HSV is established in sensory ganglia when genomic viral DNA is transported to the nuclei of sensory neurons. The viral DNA is maintained throughout the life of the neuron in a partially heterochromatic state. Replication initiates from some of these genomes periodically, and infectious virus is delivered down the axon to the original site of epithelial infection, causing recurrent disease. Specific cleavage or an induced lethal mutation of latent viral DNA would potentially preclude recurrent infections, thus curing patients of HSV infection for the first time. Such an advance would revolutionize treatment. Although it is still in the early stages, use of endonuclease systems such as CRISPR/Cas9 to target herpesvirus genomes in infected cells is ongoing ^45^. The delivery of these systems to edit viral genomes within neurons *in vivo* will pose a challenge, but use of adeno-associated viruses or other neurotropic vectors represents a promising approach ^26^.

### Repression of cellular targets

Intriguing studies suggest that antiviral targets might include cellular genes. Repurposing of drugs with known activities therefore has the potential to broadly enrich the anti-HSV pharmacopeia. For example, inhibition of the AKT signaling pathway can limit HSV replication in the eye ^46^. As another example, because reactivation from latent infection requires that the viral genome convert from a heterochromatic to a euchromatic state, drugs that preclude this conversion have the potential to prevent recurrent disease ^47, 48^.

## Abbreviations

CNS, central nervous system; CSF, cerebrospinal fluid; HSV-1, herpes simplex virus type 1; HSV-2, herpes simplex virus type 2; PCR, polymerase chain reaction; SEM, disease localized to the skin, eye, and mouth
